# The social media scale for depression in adolescence

**DOI:** 10.1080/02673843.2025.2450425

**Published:** 2025-01-15

**Authors:** Eve Twivy, Daniel Freeman, Ciorsdan Anderson, Bao Sheng Loe, Felicity Waite

**Affiliations:** a Department of Experimental Psychology, University of Oxford, Oxford, UK; b Oxford Institute of Clinical Psychology Training and Research, University of Oxford, Oxford, UK; c Oxford Health NHS Foundation Trust, Oxford, UK; d The Psychometrics Centre, University of Cambridge, Cambridge, UK

**Keywords:** Social media, depression, wellbeing, adolescence, measure development

## Abstract

Social media forms a significant part of adolescents’ lives, yet its impact on depression is unclear. We aimed to develop a questionnaire assessing different ways of using social media, and use it to understand potential associations with depression in adolescence. One thousand one hundred and forty adolescents completed an item pool. Factor analyses were conducted to derive the Social Media Scale (SMS). Ant Colony Optimization was used to develop a short-form SMS containing the factors which uniquely predicted depression as determined by structural equation modelling. The 45-item, nine-factor, SMS, had an acceptable model fit. Five factors (Social comparison; Passing time; Hostility from others; Hostility towards others; and Seeking support) uniquely contributed to depression and together explained 44% of its variance. These factors formed a 15-item short-form SMS, which had an excellent model fit. Social comparison and Passing time on social media had the strongest associations with depression and may be targets for future psychological interventions.

## Introduction

### Adolescent depression

Depression is the most common mental health problem worldwide (Friedrich, 2017) and has a peak period of onset during adolescence (Hankin et al., 2015). Depression that begins in adolescence is associated with poorer long-term psychosocial outcomes (Clayborne et al., 2019). Therefore, it is important to determine which factors contribute to adolescent depression and could be potentially targeted in interventions. Social media is an integral part of adolescents’ social world (West et al., 2021); however, current understanding of its impact on depression is limited.

### Social media

Social media is an umbrella term for applications and websites that allow people interact online. The social world gains increased importance as adolescents develop their independence and identity (Rapee et al., 2019), with estimates that 97% of teenagers use social media (Pew Research Centre, 2018). Social media is frequently cited as a cause for the rise in mental health problems in adolescents (Twenge et al., 2018); however, the evidence is inconclusive (Odgers & Jensen, 2020). Systematic reviews have identified cross-sectional associations between social media use and adolescent depression, yet the effects are small and inconsistent (Keles et al., 2020; McCrae et al., 2017). Most studies have assessed quantity of use, such as time spent online (Granic et al., 2020; Odgers & Jensen, 2020); thus, there is minimal understanding regarding the nature of online interactions.

In qualitative studies, young people have expressed that social media is a double-edged sword (O’Reilly et al., 2018; Radovic et al., 2017; Singleton et al., 2016; Twivy et al., 2023). There have been calls for research to investigate the positive and negative ways that adolescents use social media and how this impacts on mental health and wellbeing (Orben et al., 2020; Piteo & Ward, 2020; Prinstein et al., 2020). Young people have also identified this as a priority research area (Foundation, 2021). Overall, the field would benefit from a more nuanced exploration of social media use informed by an understanding of psychological theory.

### Psychological models of depression

The predominant psychological understanding and treatment of adolescent depression draws on cognitive and behavioural models (Bernaras et al., 2019; National Institute for Health and Care Excellence, 2019; Weersing et al., 2017). The interaction of adverse life events and negatively biased processing of information is proposed to increase vulnerability to developing depression (Lakdawalla et al., 2007). Core maintaining factors include negative thoughts about the self, world, and future, rumination, inactivity, and unhelpful coping behaviours (A. T. Beck, 1979; Moorey, 2010; Verduyn et al., 2009). Whilst research indicates that cognitive and behavioural processes play a role in adolescent depression in an offline context, there is a need to consider how they play out in the online social world.

### Potential psychological processes involved in social media use

Negative self-beliefs, which characterize depression, could be exacerbated by comparing one’s attributes to others on social media. Social comparison theory (Festinger, 1954) proposes that upward social comparisons (i.e. comparisons to people perceived to be better than you) can lead to negative self-beliefs. Social comparisons are common during adolescence (Rapee et al., 2019), and social media provides opportunities for upward comparisons as people tend to present their best selves online. A prior study found that social comparison on social media was associated with adolescent depression (Nesi & Prinstein, 2015).

Another way in which negative self-beliefs might interact with social media use is via impression management (i.e. actions taken to carefully present yourself, in order to control how you are perceived by others). Attempts to hide the true self could be conceptualized as safety-seeking behaviours (Salkovskis, 1991), which prevent disconfirmation of negative self-beliefs. Social media provides opportunities for impression management due to the lack of immediate response required and technology features, such as photo filters. However, this has not been studied empirically in the context of adolescent depression.

Social media could also be used in ways that resemble unhelpful coping behaviours in depression (i.e. ineffective attempts to cope with distress or counteract negative beliefs; J. S. Beck, 1995; Moorey, 2010). A qualitative study found that adolescents with depression used social media as a coping tool when feeling low, such as oversharing information or ‘ranting’ (Radovic et al., 2017). These strategies could lead to unintended negative consequences, such as judgement from others, and thus may exacerbate low mood.

Another psychological process that might occur within the context of social media use is rumination (i.e. repetitive negative past-oriented thinking; Nolen-Hoeksema, 1991). The ambiguous nature of online interactions, due to limited non-verbal cues, may trigger rumination. Preliminary evidence indicates that rumination about social media is associated with psychological distress (Parris et al., 2020), however further research is needed. A related concept of rumination is worry (i.e. repetitive negative future-oriented thinking), which has also been associated with adolescent depression (Blain-Arcaro & Vaillancourt, 2016; Danielsson et al., 2013). Delays between posting online and gaining a response may trigger worry. Adolescents with mental health difficulties have reported fears that bad things will happen on social media (Calancie et al., 2017; Singleton et al., 2016), but this requires quantitative investigation.

A protective psychological process that might be fostered on social media is social connectedness. This is thought to create a sense of belonging, which is considered a fundamental human need (Maslow, 1943). Psychological models of wellbeing, such as PERMA theory, include connectedness as a central component (Seligman, 2018). Social connectedness is also considered a protective factor against depression in adolescents (McLoughlin et al., 2019). The potential benefits of connecting with others on social media, given the constant availability and access to wider communities, were highlighted during the COVID-19 pandemic (Hamilton et al., 2021; Magson et al., 2021).

### Aim

The aim of this study was to develop a questionnaire assessing different ways of using social media, and use it to understand potential associations with depression in adolescence. Despite the dominant discourse about social media negatively impacting adolescent depression, closer inspection of the evidence base reveals a number of inconclusive findings. Prior research has primarily focused on measuring time spent on social media, rather than exploring the different ways that adolescents engage with social media. Given the lack of existing measures, there was a need to develop a self-report questionnaire assessing positive and negative behaviours and thoughts occurring in the context of social media use. Subsequently, by determining whether there are relationships between particular aspects of social media use and adolescent depression, it could provisionally indicate ways of using social media that are clinically relevant. Given that mental wellbeing is different to the absence of mental ill health (Keyes, 2013), exploring the relationship between aspects of social media use and adolescent wellbeing could provisionally identify any potential benefits – in addition to drawbacks – of social media.

### Objectives

The primary objectives of this study were to (1) develop a multi-dimensional measure of social media use by adolescents and assess its psychometric properties, and (2) determine the aspects of social media use that may be associated with depression in adolescence. A secondary objective was to explore the associations of social media use with psychological wellbeing.

## Methods

### Design and procedure

This study was an exploratory cross-sectional study. Data were collected via an online survey hosted by Qualtrics XM platform.

### Ethics

This study received Health Research Authority (HRA) and Health and Care Research Wales (HCRW) approval (Reference: 21/LO/0775). In accordance with guidelines for online research (Authority, 2018; British Psychological Society, 2013), informed consent was obtained via an information sheet and checkboxes. Participants ≥16 years provided consent. Participants <16 years provided assent, in addition to parental consent.

### Participants

Participants were 1140 adolescents, which exceeded the minimum 5:1 participant-to-variable ratio required for the factor analyses (Gorsuch, 1983; Stevens, 1996). Inclusion criteria were being aged 11–18 years, living in the United Kingdom, using social media, and enrolment at secondary school or sixth form college. Exclusion criteria was a self-reported diagnosis of a moderate or severe learning disability. Participants were recruited between December 2021 and March 2022 through social media adverts, schools, charities, and Child and Adolescent Mental Health Services (CAMHS). Most participants (81.1%) were recruited via social media, with schools being the second-largest source of participants (11.8%).

### Measures

#### Demographics

Demographic variables were obtained, including age, school year, gender, and ethnicity.

#### Revised Children’s Anxiety and Depression Scale (RCADS)

Depression and anxiety were measured using the RCADS (Chorpita et al., 2000). This is a 47-item self-report measure with the frequency of symptoms reported on a scale from 0 (never) to 3 (always). Good psychometric properties of the RCADS have been reported in both clinical and non-clinical groups (Chorpita et al., 2005; Donnelly et al., 2018).

#### EPOCH measure of adolescent wellbeing

Wellbeing was measured using the EPOCH Measure of Adolescent Well-being (Kern, Benson, Steinberg, & Steinberg, 2016). This 20-item self-report measure captures positive psychological characteristics. Statements are rated on a scale of 1 (almost never/not at all like me) to 5 (almost always/very much like me), and a total score was calculated. This measure has been validated with participants aged 10–18-year-olds, and has adequate psychometric properties.

#### Social media scale (SMS)

The SMS tried to capture motivations, behaviours, thoughts, and experiences related to social media use that potentially had theoretical relevance to adolescent depression. The item pool comprised 93 items (see S1), which were rated on a frequency scale of 0 (none of the time) to 4 (all the time). Seventy items were developed by the authors and the rest originated from three existing social media measures (Bird et al., 2018; Parris et al., 2020; Warnock-Parkes & Clark, Unpublished).

#### Item development

Consistent with measure development guidance (Boateng et al., 2018) complementary methods were used to identify domains to incorporate in the SMS. First, cognitive-behavioural models of adolescent depression were reviewed (Bernaras et al., 2019; Lakdawalla et al., 2007). Secondly, a review was conducted of nine qualitative studies on young people’s perspectives of social media and how it influences their mental health (Baker et al., 2019; Burnette et al., 2017; Calancie et al., 2017; MacIsaac et al., 2018; O’Reilly, 2020; O’Reilly et al., 2018; Radovic et al., 2017; Scott & Woods, 2019; Singleton et al., 2016). Thirdly, a focus group was held with four adolescents (aged 17–18 years) with lived experience of depression to explore how social media impacts on their mood. Finally, opinions of clinicians and researchers in the field were obtained to ensure that the domains covered aspects of social media use that they would find useful in an assessment tool.

By applying psychological theory to the qualitative information gathered, the following cognitive and behavioural processes were identified that might occur within the context of social media use: social comparison, impression management, unhelpful coping strategies, rumination, worry, and connecting with others. Motivations for using social media (i.e. avoiding exclusion, fear of missing out, connection, coping, and pleasure) and experiences on social media (i.e. negative feedback from others) were also deemed important to provide context to these processes. The generation of items for each theorized domain (see S1) was informed by qualitative accounts of adolescents, and existing measures of social media use and psychological processes ‘offline’. Finally, five adolescents with lived experience of depression reviewed the items to ensure relevance and readability.

#### Data analysis

Data from the SMS was treated as a continuous variable across the analyses (Norman, 2010; Robitzsch, 2020; Zumbo & Zimmerman, 1993). Factor analysis of the SMS was conducted in JASP (Version 0.16.1). Prior to this, the sample was divided to form a derivation sample and a validation sample using random case selection in SPSS (Version 27.0.1.0). After checking Bartlett’s Test of Sphericity and the Kaiser-Meyer Olkin Measure of Sampling Adequacy (KMO), exploratory factor analysis (EFA) was conducted to determine the underlying factor structure in the derivation sample (Fabrigar & Wegener, 2011). The estimation method was principal axis factoring with promax rotation, because some factors were expected to be related (Kahn, 2006). Items were removed before EFA if bivariate correlations between items were very high (*r* > .85) or very low (*r* <.3; Yong & Pearce, 2013). The number of factors retained was determined by parallel analysis and the requirement for there to be at least three items per factor (Knekta et al., 2019). Items were removed during EFA to obtain a clean structure if none of their factor loadings were >0.4 or if they had cross-loadings of >0.4 across multiple factors. The analysis was repeated each time items were removed. Subsequently, items were considered for removal for content reasons (i.e. awkward wording, mismatch with construct, or redundancy).

Using the validation sample, confirmatory factor analysis (CFA) was conducted to assess the factor model arising from EFA (Fabrigar & Wegener, 2011). The estimation method was robust maximum likelihood (MLR) due to having non-normal data, thus robust chi-square values were reported (Brown, 2015). Items with factor loadings <0.4 were removed, and modification indices were examined (Brown, 2015). Acceptable model fit was determined by a Comparative Fit Index (CFI) and Tucker Lewis Index (TLI) above 0.9 (Awang, 2012; Hoyle, 1995), a Root Mean Square Error of Approximation (RMSEA) below 0.08 (Browne & Cudeck, 1993), and a Standardized Root Mean Square Residual (SRMR) below 0.08 (Hu & Bentler, 1999).

The internal consistency of the factors derived from CFA was measured using Cronbach’s alpha, whereby α ≥ .7 was considered acceptable (Taber, 2018). The factor scores were obtained using the regression method (DiStefano et al., 2009) in R (Version 4.2.0).

To determine the association between the SMS factors and depression and wellbeing, Pearson correlations were conducted in JASP (Version 0.16.1). Structural equation modelling (SEM), using backward elimination, was also conducted to determine the social media factors most predictive of depression. The SMS factors retained in SEM were then used to create a short-form SMS.

Ant Colony Optimization (ACO), as described by Olaru et al. (2019), was used to create a short-form version of the SMS with factors that uniquely predict depression. ACO selects items that optimize the psychometric properties of a scale. The psychometric properties specified were model fit (i.e. CFI ≥ 0.90, RMSEA ≤ 0.06, and SRMR ≤ 0.06) and reliability (i.e. Omega ≥ .7). It was specified that 3 items per factor should be retained to obtain a multifactorial 15-item scale. This decision was guided by the aim of creating a quick-to-administer questionnaire that retained adequate reliability and breadth of item content within each social media factor. ACO, using robust maximum likelihood estimation, was performed with a maximum of 30 iterations and 50 ants per iteration. Five runs of ACO were performed to compare the models and obtain the best solution (Olaru et al., 2019). ACO was performed on the whole sample to optimize the algorithm’s capabilities of estimating the targeted model parameters. The whole dataset (*n* = 1140) was subsequently split randomly into two sub-samples (*n* = 570) to assess measurement invariance for the final selected items. Measurement invariance is used to determine whether the questionnaire measures the same constructs in different groups. It involved checking that the model fit indices did not differ significantly as the estimated model parameters (i.e. factor loadings, item intercepts, and item residuals) were constrained to be the same across the sub-samples.

## Results

### Participant characteristics

One thousand one hundred and forty-one participants (aged 11–18 years) completed the SMS. One participant was excluded due to invalid survey responses, resulting in 1140 including participants. Of these participants, 1102 completed the RCADS and 1080 completed the EPOCH measure. Demographic and clinical characteristics are presented in Table 1. The participants were largely female and of White ethnicity.

### Development of the SMS

EFA using the derivation sample (*n* = 570) was suitable because Bartlett’s test of sphericity was significant (X^2^ = 29756.12, *df* = 4005, *p* < .001) and the overall KMO test for sampling adequacy was high (KMO = 0.94; Yong & Pearce, 2013). Three items were deleted prior to EFA: one item (‘I am not as attractive as other people’) had high correlations with similar items, and two items (‘I post anonymously’, ‘I filter who can see my posts’) had low correlations with all items.

Parallel analysis initially suggested 12 possible factors; however, after the removal of items during EFA, the parallel analysis suggested nine factors. A total of 44 items were deleted during EFA: 26 items had low factor loadings, five items had cross-loadings, five items were redundant, five items were theoretically inconsistent with other items in the factor, and three items had awkward wording. EFA of the remaining 46 items resulted in a nine-factor model, which explained 55% of the variance. The factors were labelled as follows: Impression management, Hostility from others, Social comparison, Fear of social exclusion, Pleasure, Connecting with others, Passing time, Seeking support, and Hostility towards others. Factor loadings are in Table 2. Correlations between factors did not indicate a higher-order model (see S2).

CFA, using the validation sample (*n* = 570), was conducted using the nine-factor model derived from EFA. One item (‘I ask others to tell me that everything will be okay’) was deleted due to a low factor loading. After examining the modification indices, the residual correlations of three pairs of items were added into the model. The items were deemed to be clinically important to retain in the questionnaire, and there were conceptual reasons that might account for why these items shared variance beyond that explained by the model: ‘I don’t look as good as other people’ and ‘My body shape isn’t as nice as other people’ both captured appearance-related aspects of social comparison, ‘I am not as likeable as other people’ and ‘I’m not as funny as other people’ both captured personality-related aspects of social comparison, and ‘I make a special effort with my appearance for photos/videos that may be put online’ and ‘I retake photos multiple times before posting or sending’ both related to preparation of visual content as a form of impression management. The resulting model had an acceptable fit (χ2 = 1954.84, *df* = 906, *p* < .001, CFI = 0.911, TLI = 0.903, RMSEA = 0.045, SRMR = 0.054). Factor loadings are in Table 2, and factor correlations are in S3. All factors had correlations < 0.85, thus indicating discriminant validity between factors (Kline, 2011). See S4 for the final 45-item SMS.

The internal consistency of the factors, assessed across the whole sample, ranged from acceptable to excellent: Seeking support (α = .70), Connecting with others (α = .73), Hostility towards others (α = .76), Passing time (α = .78), Pleasure (α = .81), Fear of social exclusion (α = .84), Social comparison (α = .90), Hostility from others (α = .92), and Impression management (α = .92).

### Associations of SMS with depression and wellbeing

Table 3 displays the correlations between the scores on the 45-item 9-factor SMS with depression and well-being measures. All SMS factors, aside from Pleasure and Connecting with others, were significantly and positively correlated with depression. Social comparison and Passing time had the largest effect sizes. Pleasure and Connecting with others were significantly and positively correlated with wellbeing. All other SMS factors, aside from Seeking support, were significantly and negatively correlated with wellbeing.

The seven SMS factors which had a significant bivariate correlation with depression were regressed onto the depression factor in a single structural equation model. All factors were significant predictors; however, due to suppressor effects two factors (Impression management and Fear of social exclusion) were removed. The suppressor effect was identified as the factors had a negative coefficient in the SEM, whilst their bivariate correlations with depression were positive. Social comparison, Passing time, Seeking support, Hostility from others and Hostility towards others all uniquely and significantly contributed to depression (see Table 4). The model fit was acceptable (χ2 = 4256.971, *df* = 1386, CFI = 0.914, TLI = 0.908, RMSEA = 0.043, SRMR = 0.051). The final SEM model explained 44% of the variance in depression.

### Development of the short-form SMS

Using the five SMS factors retained in the SEM, a 15-item short-form SMS was created using ACO (see S5). Across five runs of ACO, the same model was obtained, suggesting that this is a robust solution. The final selected items are displayed in Table 5 with the factor loadings.

The results showed excellent model fit (CFI = 0.984, RMSEA = .030, SRMR = 0.031) and each social media factor had at least adequate reliability (≥.70). The measurement invariance statistics (see Table 6) indicate that the model is invariant at the strict level for the two randomly assigned sub-groups. The model fit of the strict model (i.e. factor loadings, item intercepts and item residuals were constrained to be equal across subgroups) was very similar (ΔCFI ≤0.01) to the model fit of the configural model (i.e. no constraints on the parameters). This suggests that the questionnaire works reliably across separate samples.

## Discussion

In the current study, we developed a novel social media scale, and tested its associations with adolescent depression and wellbeing. The SMS had good psychometric properties. The underlying constructs within the SMS included motivations for using social media (Passing time, Fear of social exclusion, and Pleasure) in addition to activity on social media (Social comparison, Impression management, Hostility towards others, Hostility from others, Connecting with others, and Seeking support).

Pleasure and Connecting with others were the only factors that were not individually associated with depression, however they were positively associated with wellbeing. Five factors (Social comparison; Passing time; Hostility from others; Hostility towards others; and Seeking support) uniquely contributed to depression and had good explanatory power. These factors were used to develop a short-form SMS with excellent psychometric properties. Due to the likely shared variance with other social media factors (Maassen & Bakker, 2001), Fear of social exclusion and Impression management were not included in the short-form SMS. These factors may be less important in explaining depression when measured in conjunction with other factors. Nevertheless, these factors may be clinically relevant to measure, particularly in the context of comorbid mental health difficulties. Overall, the long-form SMS has utility for broader investigations of the positive and negative effects of social media use on mental wellbeing and mental ill health, whilst the short-form SMS has specific relevance to adolescent depression.

### Motivations for using social media

Adolescent depression was associated with using social media out of fear of social exclusion and to pass time. Passing time was one of the strongest contributors to depression out of the social media factors. This aligns with previous research finding associations of boredom with depression (Fahlman et al., 2009; Verduyn et al., 2009) and supports emerging evidence that the motivations behind social media use are important in the context of adolescent mental health (Barry et al., 2017; Keles et al., 2020; Stockdale & Coyne, 2020).

There was no association between using social media for pleasure and depression. This was unexpected given that anhedonia (i.e. lack of pleasure) is a feature of depression (A. T. Beck, 1979; Moorey, 2010). However, more recent findings suggest that anhedonia may be less central to adolescent depression compared to adult depression (Rice et al., 2019; Twivy et al., 2023). Importantly, using social media for pleasure was positively associated with adolescent wellbeing. This is aligned with the PERMA theory that positive emotions contribute to wellbeing (Seligman, 2018), and qualitative accounts of adolescents about the benefits of social media (Radovic et al., 2017).

### Activity on social media

Social comparison was one of the strongest contributors to adolescent depression. This builds on previous research with adults (Appel et al., 2016) and adolescents (Hjetland et al., 2024; Nesi & Prinstein, 2015) to highlight the pertinence of online social comparison to depression. A further finding was that adolescents with depression reported more frequent impression management on social media. This was predicted theoretically, and is consistent with emerging evidence on the significance of digital self-presentation on adolescent mental health (Hjetland et al., 2024; Skogen et al., 2021). Whilst causal inferences cannot be drawn in this study, it is possible that both social comparison and impression management have a bi-directional relationship with negative self-beliefs in the context of depression.

Adolescent depression was associated with more hostile interactions on social media, which included greater hostility towards others. Indeed, previous research has found that depression is associated with increased aggression towards others (Dutton & Karakanta, 2013), which may be a coping strategy. This study’s findings indicate that expressions of hostility may extend to social media. This study also found that greater hostility from others was associated with higher levels of depression. This is in accordance with substantial research associating cyberbullying and cybervictimization with increased depressive symptoms (Nixon, 2014; Reed et al., 2016; Skogen et al., 2023). Prospective studies are still required to determine the direction of this relationship (Kaltiala-Heino & Fröjd, 2011; Nixon, 2014).

Connecting with others on social media appeared to be unrelated to adolescent depression in this study. This was unexpected given that depression has been associated with social withdrawal (Moorey, 2010); however, this may not extend to social media interactions. Nevertheless, connecting with others was positively associated with adolescent wellbeing, as predicted by PERMA theory (Seligman, 2018) and qualitative accounts of adolescents (Radovic et al., 2017). Another social interaction which did have an association with depression was seeking support on social media. Higher levels of depression were associated with increased levels of support seeking. It would be interesting for future research to consider the outcomes of seeking support to determine whether this leads to positive or negative outcomes.

## Limitations

There were limitations with the evaluation of the study measures. Regarding the SMS, test–retest reliability data were not collected and convergent validity was not determined. Additionally, some of the psychological processes (i.e. rumination and worry) that we aimed to capture in the SMS did not emerge as distinct constructs. Moreover, exposure to harmful content online through unwanted social media algorithms (Costello et al., 2023) or unwanted attention (Skogen et al., 2023) is beyond the scale’s scope. Therefore, the SMS may not capture all aspects of social media that are relevant to adolescent depression.

The findings may have limited generalizability to younger adolescents, given that most participants were >15 years. Recruitment of younger adolescents was likely impacted by the requirement for parental consent, and official age restrictions on social media sites. A meta-analysis found that the peak age at onset of depressive symptoms is 15.5 years (Solmi et al., 2022); therefore, the study participants represents a group for which the SMS is likely to be particularly useful. The factors within the SMS are anticipated to be applicable to younger adolescents given that relevant psychological processes, such as social comparison and impression management, emerge during childhood (Aloise-Young, 1993; Eccles, 1999). However, the nature of social media use and its impact might vary according to stage of adolescent development (Orben et al., 2022). For example, the onset of puberty may give rise to more appearance-related concerns (Markey, 2010) on social media, and more developed impulse control in later adolescence (Meeus et al., 2021) could result in more purposeful use of social media. The SMS would benefit from a validation in a larger group of 11–15-year olds. Subsequently, age differences in the role of different social media factors in depression could be explored. Opt-out parental consent procedures may facilitate recruitment in younger adolescents (Harris & Porcellato, 2018; Tigges, 2003).

Another notable characteristic of the study participants is that the majority (83%) were White. However, this is representative of the United Kingdom population, which is 87% White (Office for National Statistics, 2013). Furthermore, a recent study found that the type of social media use in adolescents did not differ by ethnicity (Winstone et al., 2022); therefore, this may not have impacted the study findings.

## Conclusions

Social media is an important part of adolescents’ lives. This study developed a novel, psychometrically robust questionnaire – the SMS – to assess the ways that adolescents engage with social media. A short-form SMS was also developed which is comprised of aspects of social media use most related to adolescent depression. Prior research has predominantly measured time spent online; however, this has prevented exploration of a more nuanced relationship between social media use and depression. To illustrate, in this study, adolescent depression was not associated with increased frequency of all types of social media use. Connecting with others and Pleasure were not associated with depression but were associated with higher wellbeing. Therefore, blanket interventions like ‘reducing screen time’ are unlikely to be the solution and could negatively impact on adolescent wellbeing. The associations of negative aspects of social media use, especially using social media to pass time and engaging in social comparison, with depression provide preliminary indications that aspects of social media use play a role in adolescent depression. The potential for specific types of social media use to exacerbate depression warrants further investigation in longitudinal and experimental studies as these may be fruitful targets for psychological interventions.

## Supplementary Material

Supplemental Material

## Figures and Tables

**Table 1:** Participant characteristics.

Characteristic	Total sample (*n* = 1140)
**Age**	**Mean (*SD*)** 16.3 (1.1)
**Gender**	**n(%)**
Female	709 (62.2)
Male	339 (29.7)
Other (including non-binary, gender fluid, transgender, and agender)	92 (8.1)
**Ethnicity**	**n (%)**
White	941 (82.5)
Mixed or Multiple	72 (6.3)
Asian	93 (8.2)
Black, Caribbean or African	19 (1.7)
Other	15 (1.3)
**Currently receiving mental health services**	**n (%)** 360 (31.6)
**RCADS Scores^a^**	**Mean (SD)**
Depression	17.6 (7.5)
Anxiety	55.7 (23.8)
**EPOCH Scores**	53.9 (14.3)

a Raw scores were used because T-scores of adolescents with gender identities other than male or female could not be determined due to a lack of normative data on the RCADS for gender minority groups.

**Table 2:** Factor loadings from EFA and CFA on the SMS.

Factors	Items	EFA loadings	CFA loadings
Impression management	I reword messages or posts multiple times.	0.450	0.469
	I make a special effort with my appearance for photos/videos that may be put online	0.953	0.704
	I retake photos multiple times before posting or sending.	1.003	0.747
	I edit, manipulate or use filters on photos/snaps.	0.503	0.525
	I get my friend’s opinion on my photos before posting.	0.460	0.566
	I make an effort to come across well.	0.610	0.501
	I try to look perfect.	0.779	0.757
	I try to picture how I appear to others on social media.	0.729	0.774
	I check how others respond to my posts.	0.728	0.683
	Worries that people will judge my posts pop into my mind.	0.616	0.765
	I worry that I won’t look attractive in social media posts.	0.657	0.816
	I keep thinking about how other people have reacted to my posts.	0.629	0.743
Hostility from others	I get nasty comments from others on social media.	0.798	0.819
	People send me threats on social media.	0.775	0.764
	People criticize me on social media.	0.872	0.866
	People make fun of me on social media.	0.906	0.890
	I get bullied on social media.	0.767	0.823
Social comparison	I don’t look as good as other people.	0.719	0.814
	My body shape isn’t as nice as other people.	0.719	0.753
	Other people have better social lives than me.	0.597	0.793
	I am not as likeable as other people.	0.902	0.812
	I’m not as funny as other people.	0.768	0.724
Fear of social exclusion	To make sure I am not left out	0.629	0.723
	To fit in at school/college.	0.841	0.817
	To be accepted by others.	0.788	0.860
	To make sure I don’t miss out on anything.	0.649	0.622
Pleasure	To have fun.	0.705	0.765
	To cheer me up.	0.677	0.706
	To keep up my interests.	0.689	0.607
	To have a laugh.	0.717	0.740
Connecting with others	I keep in touch with friends or family.	0.598	0.557
	I reconnect with people who I lost touch with.	0.447	0.440
	I share memes, pictures or videos with friends or family.	0.587	0.648
	I make plans on social media to meet up with friends.	0.681	0.585
	I joke around with friends	0.611	0.698
Passing time	To fill my time when I am bored or unmotivated.	0.716	0.696
	To distract myself from what is going on around me.	0.646	0.712
	To pass the time when I can’t sleep.	0.598	0.699
	I spend hours scrolling through social media.	0.571	0.711
Seeking support	To get support.	0.772	0.593
	I seek support from people who I can rely on.	0.603	0.731
	I connect with people who have been through similar experiences.	0.549	0.612
	I ask others to tell me that everything will be okay.	0.490	–
Hostility towards others	I start fights or arguments.	0.604	0.791
	I am aggressive towards others.	0.750	0.844
	I purposefully upset others.	0.742	0.688

**Table 3:** Bivariate correlations between the SMS factors and depression and wellbeing.

45-item SMS Factors	Depression	Wellbeing
Impression management	.438*	−.257*
Hostility from others	.328*	−.224*
Social comparison	.560*	−.431*
Fear of social exclusion	.259*	−.151*
Pleasure	−.052	.257*
Connecting with others	−.014	.250*
Passing time	.521*	−.332*
Seeking support	.252*	.054
Hostility towards others	.297*	−.241*

* *p* < .001; *r* = .1–.3 is a small effect size, *r* = .3–.5 is a medium effect size, *r* = .5–1.0 is a large effect size.

**Table 4:** Regression coefficients for the depression structural equation model.

Predictors	B	Standard error	*p*-value	Standardised coefficient
Hostility from others	0.067	0.030	.024	.070
Social comparison	0.272	0.028	<.001	.420
Passing time	0.266	0.065	<.001	.199
Seeking support	0.104	0.034	.002	.099
Hostility towards others	0.086	0.033	.010	.085

**Table 5:** Factor loadings in the ACO model.

Factors	Items	Loadings
Hostility from others	I get nasty comments from others on social media.	0.766
	People criticize me on social media.	0.899
	People make fun of me on social media.	0.906
Social comparison	I don’t look as good as other people.	0.808
	Other people have better social lives than me.	0.799
	I’m not as funny as other people.	0.726
Passing time	To fill my time when I am bored or unmotivated.	0.645
	To pass the time when I can’t sleep.	0.674
	I spend hours scrolling through social media.	0.756
Seeking support	To get support.	0.670
	I seek support from people who I can rely on.	0.679
	I connect with people who have been through similar experiences.	0.639
Hostility towards others	I start fights or arguments.	0.729
	I am aggressive towards others.	0.805
	I purposefully upset others.	0.674

**Table 6:** Measurement invariance testing for the ACO model.

	df	X^2^	CFI	RMSEA	SRMR
Configural	160	273.615	0.982	0.035	0.037
Metric	170	293.631	0.981	0.036	0.039
Scalar	180	299.832	0.981	0.034	0.040
Strict	195	311.650	0.982	0.032	0.039

## Data Availability

The participants of this study did not give consent for their raw data to be shared publicly.
