# Supplementary material for: The social media scale for depression in adolescence
Source: Int J Adolesc Youth. Author manuscript; Available in PMC 2025 Dec 31. (PMC7617924; doi:10.1080/02673843.2025.2450425)
Supplement: Supplemental Material [file EMS206872-supplement-Supplemental_Material.pdf]

S1. Original Items in the SMS

Item	Theorised domain
Please select how often you use social media for each of the following reasons.	
To make sure I am not left out.<br>To avoid being rejected by others.<br>To fit in at school/college.<br>To be accepted by others.	Avoiding exclusion
To make sure I don't miss out on anything.<br>To keep up to date with what people are doing.<br>To check if friends are having fun without me.<br>To keep up with trends.	Fear of missing out
To connect with others.<br>To feel less lonely.<br>To share information or experiences with others.<br>To help others.	Connection
To get support.<br>To help cope with negative feelings.<br>To fill my time when I am bored or unmotivated.<br>To distract myself from what is going on around me.<br>To pass the time when I can't sleep.<br>To freely express myself.	Coping
To have fun.<br>To cheer me up.<br>To keep up my interests.<br>To have a laugh.	Pleasure
Please select how often you do each of the following things when using social media.	
I think of something to post or message but decide not to.<br>I reword messages or posts multiple times.<br>I carefully select words or content that I add to posts/messages/voice-notes/videos.<br>I make a special effort with my appearance for photos/videos that may be put online.<br>I retake photos multiple times before posting or sending.<br>I edit, manipulate or use filters on photos/snaps.<br>I get my friends's opinion on my photos before posting.<br>I follow less people than follow me to appear popular.<br>I check photos that I am tagged in by others.<br>I post anonymously.<br>I try to appear funny, unique, or interesting.<br>I try to keep a low profile and not attract attention.<br>I avoid posting anything too personal about myself.<br>I exaggerate something to appear better to others.<br>I make an effort to come across well.<br>I filter who can see my posts.<br>I try to appear normal.<br>I try to look perfect.<br>I try to picture how I appear to others on social media.<br>I check how others respond to my posts.<br>I delete photos that don't get enough likes.	Impression management
Please select how often you do each of the following things on social media.	

I keep in touch with friends or family.<br>I communicate with people who I can't see in person.<br>I connect with online groups which share a common interest or identity.<br>I reconnect with people who I lost touch with.<br>I share memes, pictures or videos with friends or family.<br>I make plans on social media to meet up with friends.<br>I seek support from people who I can rely on.<br>I support other people.<br>I share articles and ideas.<br>I joke around with friends.<br>I connect with people who have been through similar experiences.<br>I find out what friends have been up to.<br>I start new relationships.	Connecting with others
I post about my feelings to get a reaction from others.<br>I post revealing photos to be noticed.<br>I post about risky behaviours (e.g. taking drugs, drinking, fighting, self-harm, stunts).<br>I ask others to tell me that everything will be okay.<br>I rant about having a bad day.<br>I start fights or arguments.<br>I spend hours scrolling through social media.<br>I ignore messages from friends.<br>I search for triggering images or information.<br>I send nude photos to others.<br>I am aggressive towards others.<br>I purposefully upset others.<br>I overshare personal things.	Unhelpful coping strategies
Please select how often each of the following things happen to you.	
I worry that people will threaten me on social media.<br>Worries that people will judge my posts pop into my mind.<br>I keep thinking that my posts won't get enough likes.<br>I can't stop worrying that someone will post negative things about me.<br>I worry that I won't look attractive in social media posts.	Worry
I can't stop thinking about what someone posted on social media.<br>I spend time alone thinking about what my posts say about who I am.<br>I keep thinking about how other people have reacted to my posts.<br>I keep asking myself why I spend so much time on social media.<br>I keep going over online conversations in my mind.	Rumination
My posts don't get enough likes or attention.<br>I get nasty comments from others on social media.<br>I see friends having fun without me on social media.<br>People send me threats on social media.<br>People criticise me on social media.<br>People make fun of me on social media.<br>I get bullied on social media.	Negative feedback from others

Please select how often you have each of the following thoughts when using social media.	
I don't look as good as other people.	Social comparison
My body shape isn't as nice as other people.	
I am not as attractive as other people.	
Other people have better social lives than me.	
I can't be as popular as other people.	
I am not as likeable as other people.	
I'm not as funny as other people.	

S2. Factor Correlations for the SMS from EFA

Factors	1	2	3	4	5	6	7	8	9
**1.** Impression management	-								
**2.** Hostility from others	.32	-							
**3.** Social comparison	.63	.30	-						
**4.** Fear of social exclusion	.58	.24	.43	-					
**5.** Pleasure	.13	.08	-.05	-.02	-				
**6.** Connecting with others	.21	-.03	-.09	.06	.40	-			
**7.** Passing time	.59	.29	.54	.40	.26	.14	-		
**8.** Seeking support	.46	.34	.22	.27	.37	.37	.30	-	
**9.** Hostility towards others	.31	.42	.25	.15	.13	.02	.36	.20	-

Note. Correlations were calculated using Pearson's *r*.

S3. Factor Correlations for the SMS from CFA

Factors	1	2	3	4	5	6	7	8	9
**1.** Impression management	-								
**2.** Hostility from others	.34	-							
**3.** Social comparison	.76	.33	-						
**4.** Fear of social exclusion	.62	.35	.52	-					
**5.** Pleasure	-.07	-.11	-.20	-.11	-				
**6.** Connecting with others	.07	-.13	-.08	-.11	.58	-			
**7.** Passing time	.62	.29	.60	.40	.14	.16	-		
**8.** Seeking support	.23	.18	.17	.14	.39	.46	.27	-	
**9.** Hostility towards others	.20	.39	.22	.12	.05	.10	.33	.05	-

Note. Correlations were calculated using Pearson's *r*.

S4. Social Media Scale (SMS)

Introduction
This questionnaire asks about your thoughts, feelings and experiences using social media. Please read each item and select how often it applies to you. There are no right or wrong answers.

Reasons for Using Social Media
Please select how often you use social media for each of the following reasons.

1	**To make sure I am not left out.**	None of the time	Hardly ever	Some of the time	Often	All of the time
2	**To fit in at school/college.**	None of the time	Hardly ever	Some of the time	Often	All of the time
3	**To be accepted by others.**	None of the time	Hardly ever	Some of the time	Often	All of the time
4	**To make sure I don't miss out on anything.**	None of the time	Hardly ever	Some of the time	Often	All of the time
5	**To have fun.**	None of the time	Hardly ever	Some of the time	Often	All of the time
6	**To cheer me up.**	None of the time	Hardly ever	Some of the time	Often	All of the time
7	**To keep up my interests.**	None of the time	Hardly ever	Some of the time	Often	All of the time
8	**To have a laugh.**	None of the time	Hardly ever	Some of the time	Often	All of the time
9	**To fill my time when I am bored or unmotivated.**	None of the time	Hardly ever	Some of the time	Often	All of the time
10	**To distract myself from what is going on around me.**	None of the time	Hardly ever	Some of the time	Often	All of the time
11	**To pass the time when I can't sleep.**	None of the time	Hardly ever	Some of the time	Often	All of the time
12	**To get support.**	None of the time	Hardly ever	Some of the time	Often	All of the time

Behaviours on Social Media
Please select how often you do each of the following things when using social media.

13	**I reword messages or posts multiple times.**	None of the time	Hardly ever	Some of the time	Often	All of the time
14	**I make a special effort with my appearance for photos/videos that may be put online.**	None of the time	Hardly ever	Some of the time	Often	All of the time
15	**I retake photos multiple times before posting or sending.**	None of the time	Hardly ever	Some of the time	Often	All of the time
16	**I edit, manipulate or use filters on photos/snaps.**	None of the time	Hardly ever	Some of the time	Often	All of the time

17	**I get my friend's opinion on my photos before posting.**	None of the time	Hardly ever	Some of the time	Often	All of the time
18	**I make an effort to come across well.**	None of the time	Hardly ever	Some of the time	Often	All of the time
19	**I try to look perfect.**	None of the time	Hardly ever	Some of the time	Often	All of the time
20	**I try to picture how I appear to others on social media.**	None of the time	Hardly ever	Some of the time	Often	All of the time
21	**I check how others respond to my posts.**	None of the time	Hardly ever	Some of the time	Often	All of the time

Please select how often you do each of the following things on social media.

22	**I keep in touch with friends or family.**	None of the time	Hardly ever	Some of the time	Often	All of the time
23	**I reconnect with people who I lost touch with.**	None of the time	Hardly ever	Some of the time	Often	All of the time
24	**I share memes, pictures or videos with friends or family.**	None of the time	Hardly ever	Some of the time	Often	All of the time
25	**I make plans on social media to meet up with friends.**	None of the time	Hardly ever	Some of the time	Often	All of the time
26	**I joke around with friends.**	None of the time	Hardly ever	Some of the time	Often	All of the time
27	**I spend hours scrolling through social media.**	None of the time	Hardly ever	Some of the time	Often	All of the time
28	**I seek support from people who I can rely on.**	None of the time	Hardly ever	Some of the time	Often	All of the time
29	**I connect with people who have been through similar experiences.**	None of the time	Hardly ever	Some of the time	Often	All of the time
30	**I start fights or arguments.**	None of the time	Hardly ever	Some of the time	Often	All of the time
31	**I am aggressive towards others.**	None of the time	Hardly ever	Some of the time	Often	All of the time
32	**I purposefully upset others.**	None of the time	Hardly ever	Some of the time	Often	All of the time

Thoughts and Experiences on Social Media
Please select how often each of the following things happen to you.

33	**Worries that people will judge my posts pop into my mind.**	None of the time	Hardly ever	Some of the time	Often	All of the time

34	**I worry that I won't look attractive in social media posts.**	None of the time	Hardly ever	Some of the time	Often	All of the time
35	**I keep thinking about how other people have reacted to my posts.**	None of the time	Hardly ever	Some of the time	Often	All of the time
36	**I get nasty comments from others on social media.**	None of the time	Hardly ever	Some of the time	Often	All of the time
37	**People send me threats on social media.**	None of the time	Hardly ever	Some of the time	Often	All of the time
38	**People criticise me on social media.**	None of the time	Hardly ever	Some of the time	Often	All of the time
39	**People make fun of me on social media.**	None of the time	Hardly ever	Some of the time	Often	All of the time
40	**I get bullied on social media.**	None of the time	Hardly ever	Some of the time	Often	All of the time

Please select how often you have each of the following thoughts when using social media.

41	**I don't look as good as other people.**	None of the time	Hardly ever	Some of the time	Often	All of the time
42	**My body shape isn't as nice as other people.**	None of the time	Hardly ever	Some of the time	Often	All of the time
43	**Other people have better social lives than me.**	None of the time	Hardly ever	Some of the time	Often	All of the time
44	**I am not as likeable as other people.**	None of the time	Hardly ever	Some of the time	Often	All of the time
45	**I'm not as funny as other people.**	None of the time	Hardly ever	Some of the time	Often	All of the time

Interpretation of the SMS

Scale Scoring

None of the time	Hardly ever	Some of the time	Often	All of the time
0	1	2	3	4

Items by Domain

Domains	Items
Impression management	13,14,15,16,17,18,19,20,21,33,34,35
Hostility from others	36,37,38,39,40
Social comparison	41,42,43,44,45
Fear of social exclusion	1,2,3,4
Pleasure	5,6,7,8
Connecting with others	22,23,24,25,26
Passing time	9,10,11,27
Seeking support	12,28,29
Hostility towards others	30,31,32

S5. Social Media Scale (SMS) Short-Form

Introduction

This questionnaire asks about your thoughts, feelings and experiences using social media. Please read each item and select how often it applies to you. There are no right or wrong answers.

Please select how often you use social media for each of the following reasons.						
1.	**To fill my time when I am bored or unmotivated.**	None of the time	Hardly ever	Some of the time	Often	All of the time
2.	**To pass the time when I can't sleep.**	None of the time	Hardly ever	Some of the time	Often	All of the time
3.	**To get support.**	None of the time	Hardly ever	Some of the time	Often	All of the time
Please select how often you do each of the following things on social media.						
4.	**I spend hours scrolling through social media.**	None of the time	Hardly ever	Some of the time	Often	All of the time
5.	**I seek support from people who I can rely on.**	None of the time	Hardly ever	Some of the time	Often	All of the time
6.	**I connect with people who have been through similar experiences.**	None of the time	Hardly ever	Some of the time	Often	All of the time
7.	**I start fights or arguments.**	None of the time	Hardly ever	Some of the time	Often	All of the time
8.	**I am aggressive towards others.**	None of the time	Hardly ever	Some of the time	Often	All of the time
9.	**I purposefully upset others.**	None of the time	Hardly ever	Some of the time	Often	All of the time
Please select how often each of the following things happen to you.						
10.	**I get nasty comments from others on social media.**	None of the time	Hardly ever	Some of the time	Often	All of the time
11.	**People criticise me on social media.**	None of the time	Hardly ever	Some of the time	Often	All of the time
12.	**People make fun of me on social media.**	None of the time	Hardly ever	Some of the time	Often	All of the time
Please select how often you have each of the following thoughts when using social media.						
13.	**I don't look as good as other people.**	None of the time	Hardly ever	Some of the time	Often	All of the time
14.	**Other people have better social lives than me.**	None of the time	Hardly ever	Some of the time	Often	All of the time
15.	**I'm not as funny as other people.**	None of the time	Hardly ever	Some of the time	Often	All of the time
